# Stereotactic Radiotherapy for Recurrent Post-Transplant Primary Central Nervous System Lymphoma

**DOI:** 10.7759/cureus.16537

**Published:** 2021-07-21

**Authors:** Cecil M Benitez, Elham Rahimy, Neil Panjwani, Lauren S Maeda, Scott G Soltys

**Affiliations:** 1 Department of Radiation Oncology, University of California, Los Angeles, Los Angeles, USA; 2 Department of Radiation Oncology, Stanford University School of Medicine, Stanford, USA; 3 Department of Hematology and Oncology, Stanford University School of Medicine, Stanford, USA

**Keywords:** stereotactic radiotherapy (srt), post-transplant lymphoproliferative disorder (ptld), primary central nervous system lymphoma (pcnsl), external beam radiation, stereotactic radiosurgery (srs)

## Abstract

Post-transplant primary central nervous system lymphoma (PCNSL) is a rare complication of solid organ transplantation. The optimal therapy for post-transplant PCNSL is not well established and generally includes reduction of immunosuppression and chemotherapy. Progression after front-line chemotherapy is common, and whole-brain radiotherapy (WBRT) is a standard salvage treatment as there is a concern that localized treatment fields would not prevent out-of-field recurrences. However, WBRT is associated with neurotoxicity and morbidity in these patients with inherently poor prognoses. Here, we report a patient with local recurrence of post-transplant PCNSL who was treated with fractionated stereotactic radiotherapy (SRT). He had no clinical toxicity from treatment and maintained pre-treatment neurocognition and performance status. Local control was achieved for 20 months following SRT, at which point he developed an in-field recurrence. He restarted lymphoma therapy but died one month later from fungal pneumonia. For central nervous system (CNS) lymphoma, further data are needed to optimize tumor control and toxicity outcomes and identify patients in whom localized radiotherapy fields may be beneficial, avoiding the potential toxicity of WBRT.

## Introduction

Post-transplant lymphoproliferative disorders (PTLDs) are a spectrum of lymphoid neoplasms that range from benign hyperplasias to aggressive lymphomas [1,2]. Up to 3% of transplant recipients may develop PTLD, although patients with multiorgan, heart, lung, or intestinal transplants have an incidence of up to 25% [3]. Over 90% of PTLD cases are associated with Epstein-Barr virus (EBV); serum and cerebrospinal fluid (CSF) EBV levels can correlate with treatment response. Approximately 5%-15% of PTLD cases can present as primary central nervous system lymphoma (PCNSL) with no evidence of systemic disease. Post-transplant PCNSL typically develops four to five years after solid organ transplantation, is more aggressive than systemic PTLD, and is less responsive to the reduction of immunosuppressive agents [3-5].

Given the rarity and poor prognosis of post-transplant PCNSL, treatment guidelines are not well established. Current approaches include immunosuppression reduction and intensive chemotherapy such as high-dose methotrexate (HD-MTX), temozolomide, and rituximab (MTR), extrapolating from de novo PCNSL treatment regimens. Consolidation may include observation (after complete response), further chemotherapy, or whole-brain radiotherapy (WBRT). WBRT leads to favorable response rates but is associated with neurotoxicity, which can negatively impact the quality of life, especially when combined with MTR in elderly patients [6-8]. Stereotactic radiosurgery (SRS) is an intracranial radiotherapy alternative to WBRT. SRS is characterized by its high accuracy and rapid dose fall-off, minimizing the dose to normal brain, and thus reducing neurotoxicity and morbidity compared to WBRT [9]. SRS, with deferral of WBRT, is the standard of care radiotherapy technique for most patients with limited brain metastases [9]. Thus, there is interest in SRS treatment of refractory and recurrent post-transplant PCNSL. Here, we present a patient with recurrent post-transplant PCNSL (following HD-MTX and immunosuppression therapy reduction) who was treated with fractioned stereotactic radiotherapy (SRT), with the deferment of WBRT.

## Case presentation

**Figure 1:**
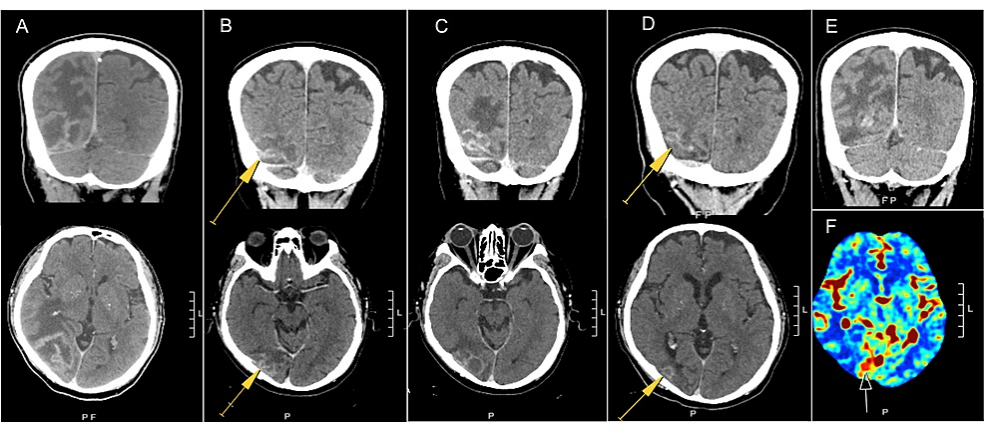
CT, coronal (top panel), and axial (bottom panel) views of the brain lesion (A) Enhancing lesion in the right parieto-occipital lobe with marked vasogenic edema at the time of initial presentation. (B) Improvement in edema and enhancement after six cycles of MTR. (C) Progression after eight cycles of MTR, with increased enhancement and vasogenic edema. (D) Two months post-radiotherapy, with minimal residual enhancement and edema. (E) Progression at 20 months post-radiotherapy, with increased enhancement (not well-shown) and vasogenic edema. (F) Axial image showing an area of increased cerebral blood volume on perfusion CT, concerning for recurrence. Yellow arrows are pointing at the treated lesion. MTR = Methotrexate, temozolomide, and rituximab; CT = computed tomography.

A 72-year-old man presented to the emergency department from his cardiology clinic with two weeks of headaches, blurry vision, imbalance, and left-sided weakness. He had a history of orthotopic heart transplant 13 years prior and kidney transplant five years prior and was maintained on mycophenolate and tacrolimus for immunosuppression. Given a magnetic resonance imaging (MRI)-incompatible pacemaker, MRI could not be obtained. Computed tomography (CT) scan of the head is shown in Figure 1. The CT scan of the head demonstrated an enhancing 4.0 cm x 3.7 cm x 3.8 cm (transverse x anterior-posterior x craniocaudal) right parieto-occipital mass expanding into the corpus callosum with mild right uncal herniation, 6 mm midline shift, and extensive vasogenic edema (Figure 1A). The lesion was suspicious for either a lymphoproliferative disorder or an immunosuppression-associated infection. He underwent stereotactic brain biopsy, which demonstrated CD20+, CD30+, BCL2+, Myc-negative, Epstein-Barr encoding region (EBER) in situ hybridization (ISH)-positive cells with a Ki-67 proliferative index of 50%. These results were consistent with monomorphic PTLD, EBV-positive diffuse large B-cell lymphoma. Positron emission tomography-computed tomography (PET-CT), slit-lamp examination, and bone marrow biopsy did not show evidence of extra-cranial or intraocular disease. Though the biopsy was EBV-positive, the patient was EBV seronegative.

In tandem with urgently starting MTR (planned for eight cycles, given every two weeks), his tacrolimus dose was reduced, mycophenolate was discontinued, and he completed a short course of dexamethasone. He experienced marked improvement of his presenting symptoms and was discharged. An interval head CT after three cycles of MTR showed that the mass was slightly decreased to 4.2 cm x 3.5 cm x 3.3 cm, and there was reduced surrounding vasogenic edema with resolved mass effect. Unfortunately, he developed acute renal failure on treatment, which delayed several cycles of HD-MTX and resulted in dose reductions of temozolomide. An interval head CT after six cycles, six weeks after the prior CT, showed continued treatment response (Figure 1B). 

Two weeks after completing the planned eight cycles of MTR, he presented to the emergency department with dizziness and headache. Head CT demonstrated mild enlargement of the residual solitary occipital mass, now 3.6 cm x 1.9 cm x 3.3 cm (previously 3.0 cm x 1.9 cm x 2.5 cm after six cycles) with associated vasogenic edema (Figure 1C). Given his age and comorbidities, his case was discussed at the interdisciplinary tumor board. WBRT was presented as an option as there were few remaining systemic options, given his transplant status, age, and comorbidities. In an effort to preserve his performance status (Karnofsky performance status, KPS, of 70%) and to avoid cognitive impairment, ultimately WBRT was not recommended. At the time of consultation, his symptoms were largely resolved on dexamethasone, and his exam was unremarkable apart from reduced bilateral peripheral vision attributed to a combination of cytomegalovirus (CMV) retinitis, diabetic retinopathy, and glaucoma. Although WBRT is the standard treatment, given that the site of his recurrent disease was the same as the site of initial presentation, a localized treatment field was thought to be reasonable to minimize the side effects of WBRT. He was aware that the risk of omitting WBRT was that the tumor may progress outside of the localized radiotherapy field. After a discussion of these risks and benefits of WBRT versus SRT to a localized field, he elected to proceed with SRT.

**Figure 2:**
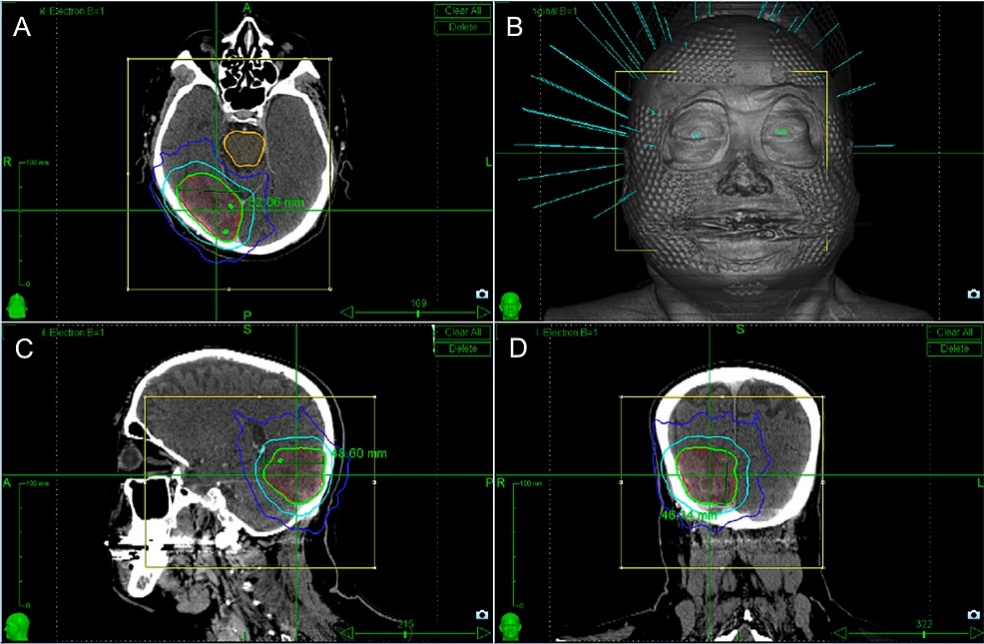
Stereotactic radiotherapy plan targeting the right occipital lymphoma (A) CT axial view of isodose lines targeting the lymphoma. (B) Head schematic with radiation beamlines shown in blue color. (C) CT sagittal view of isodose lines targeting the lymphoma. (D) CT coronal view with isodose lines targeting the lymphoma. The target volumes are shown in green color. The plan delivered 33 Gy to the 90% isodose line to the periphery of the target. The treatment was delivered over six consecutive daily fractions. Isodose lines shown in the image are detailed as follows: green is 33.0 Gy (100%), sky blue is 16.5 Gy (50%), dark blue is 8.1 Gy (approximately 25%). Orange contour outlines the brainstem. Red contour outlines the lymphoma. CT = Computed tomography.

Treatment planning was exclusively CT-based (given MRI-incompatibility of his pacemaker) and is shown in Figure 2. A custom mask was used for immobilization. The gross tumor volume (GTV) included the following: (1) the extent of tumor recurrence as defined by the post-contrast enhancement and (2) the pre-treatment extent of tumor seen at initial presentation. A 5-mm margin, without extending beyond anatomic barriers such as bone and tentorium/falx, formed the clinical target volume (CTV). With stereotactic guidance, no further PTV (planning target volume) was added. Given the large treatment volume (63 cm^3^), a hypofractionated stereotactic regimen of five days was planned; however, five days of considered stereotactic radiosurgery were not authorized by insurance, so six days of the identical treatment were authorized. The final PTV was treated to 33 Gy in six consecutive daily fractions, delivered to the 90% isodose line with a conformity index of 1.14 and a treatment time of 24 minutes per fraction. This dose was biologically equivalent to a standard WBRT dose for immunocompetent PCNSL of 45 Gy at 1.8 Gy/fraction. Pacemaker dose was monitored with in vivo dosimetry using a nanodot optically stimulated luminescence dosimeter (OSLD); the dose was low, as expected, measuring 11 cGy for the first fraction. Treatment was delivered without issues, and there were no acute or late toxicities noted.

Serial follow-up head CTs demonstrated the stability of the residual enhancement with no new lesions or edema (Figure 1D). At six months post-radiotherapy, the patient noted worsening vision and was found to have a bilateral vitreous hemorrhage, worse on the left than the right. Upon review of the radiation plan, the dose to the left eye was <1 Gy and to the right eye was <3 Gy. He was started on intravitreal bevacizumab with improvement in his vision two months later.

He was doing clinically well until 20 months following SRT, at which time he developed severe disequilibrium. Perfusion head CT demonstrated marked vasogenic edema in the right parieto-occipital lobe (Figure 1E) with increased enhancement and perfusion of the right occipital lesion (Figure 1F). Although it was uncertain if this lesion represented an in-field recurrence or radiation necrosis, he was started on dexamethasone and ibrutinib as it would treat both radiation necrosis and recurrent disease. However, before the next follow-up imaging, he was hospitalized two weeks later with multifocal fungal pneumonia. After a complicated four-day hospital course, he, unfortunately, passed away from respiratory failure. During this hospitalization, a repeat CT head demonstrated decreased edema and enhancement, most consistent with an improvement in his post-transplant PCNSL.

## Discussion

Albeit rare, post-transplant PCNSL is a known complication of solid organ transplantation and immunosuppression. A decrease in immunosuppression alone is not curative, and approximately 60% of HD-MTX responders will relapse [10]. Treatment options are extrapolated from regimens designed for de novo PCNSL. In PCNSL, WBRT in combination with HD-MTX has improved survival outcomes compared to HD-MTX [11,12] or WBRT alone [13]. Patients without prior irradiation and relapsing disease treated with WBRT have improved response rates of 60%-74% [14]. However, prognosis remains poor, and WBRT is associated with neurotoxicity and decreased quality of life [6,14,15]. Thus, WBRT is often reserved for salvage treatment. On-going clinical efforts are focusing on reducing WBRT-associated neurotoxicity while preserving the response rates.

Current approaches to reduce WBRT neurotoxicity include reducing WBRT doses with or without intensity-modulated radiation therapy (IMRT) boosts. Reducing WBRT doses to ≤24 Gy is controversial. Some studies have failed to show noninferiority of reduced doses compared to WBRT of doses ≥45 Gy, while others have shown that reduced WBRT doses may be a reasonable approach especially in patients ≥60 years old or those with complete response to HD-MTX [12,16]. Although the efficacy is not yet known, reduced WBRT doses to 30-36 Gy with IMRT boosts to the lesions for a total dose of 45 Gy may also be another reasonable approach in patients with the relapsed disease [17]. Additional studies are needed to determine the efficacy of these approaches.

Although studies are sparse, stereotactic radiosurgery (SRS) has been increasingly used as salvage therapy for patients presenting with small recurrent or refractory PCNSL. Shin et al. described 23 patients with small recurrent/refractory PCNSL (average tumor volume of 4 cm^3^) treated with single-fraction SRS (median dose of 15 Gy, range 8-20 Gy) [18]. With a median follow-up of 11 months, local control was 91% at six months and 75% at 12 months, and progression-free survival was 81% at six months and 55% at 12 months. Fourteen patients had relapsed (n = 6 out-of-field, n = 6 in-field) or refractory (n = 2) disease [18]. Further exploring the role of localized radiotherapy, a series of 55 patients treated with SRS (median 11 Gy, range 11-16 Gy) with HD-MTX in newly diagnosed PCNSL had improved tumor control and overall survival (47.6 months vs. 26.8 months) when compared to patients that received HD-MTX alone, while maintaining acceptable toxicity levels. Nine patients developed out-of-field intracranial recurrence that was treated with SRS, and no in-field recurrences were detected at a mean of 30.6 months post-SRS [19]. SRS can provide acceptable local control and utility in treated out-of-field recurrence while minimizing neurotoxicity.

Although the optimal treatment for newly diagnosed and recurrent PCNSL remains controversial, data are needed to identify patients in whom localized radiotherapy is reasonable to consider when systemic therapy options are limited and patients where a concern exists for the toxicity of WBRT, as in our patient. We report a patient with relapsed post-transplant PCNSL treated with focal SRT, who benefited from in-field tumor control for 20 months while maintaining his pre-radiotherapy neurological and performance status. Given that his tumor recurrence was at the site of the initial disease, it is unlikely that WBRT would have provided additional benefit. Although controlled for 20 months, tumor recurrence within the radiotherapy field suggests a dose greater than the equivalent of 33 Gy in six fractions may be beneficial.

## Conclusions

Given the treatment-related neurotoxicity of full-dose WBRT for the treatment of PCNSL, particularly in elderly patients, alternative radiotherapy options are needed. We present a 72-year-old man with locally recurrent post-transplant PCNSL treated with focal SRT. His intracranial tumor control for 20 months, both within and outside of the radiotherapy field, suggests that SRT may be a reasonable option in selected patients with PCNSL. Greater data are required to identify patients where tumor recurrence is likely to remain localized rather than disseminated throughout the brain and therefore in whom WBRT may be omitted.
